# Chiropractic treatment of idiopathic scoliosis with the CLEAR Institute method: a description of the protocol

**DOI:** 10.1186/1748-7161-8-S2-P6

**Published:** 2013-09-18

**Authors:** Alan Woggon, Daniel Martinez

**Affiliations:** 1 CLEAR Scoliosis Institute, St Cloud, MN, USA

## Background

Chiropractic care is a healthcare discipline that focuses upon the diagnosis and treatment of problems that affect the alignment of the muscles and bones of the body. According to the National Board of Chiropractic Examiners, 2.7 million visits are made each year in the U.S. to chiropractors for scoliosis and scoliosis-related complaints[1]. Despite the frequency with which chiropractic services are employed for scoliosis, there is insufficient evidence to draw conclusions on the chiropractic treatment of scoliosis; the majority of published papers are case reports, with the only exception being a cohort study by Lantz et al.[2] that finds little benefit to the practices commonly employed by most chiropractors in the treatment of scoliosis[3]. Recognizing this, a novel chiropractic protocol for the evaluation, assessment, and treatment of scoliosis was developed by the CLEAR (Chiropractic Leadership, Educational Advancement, and Research) Scoliosis Institute, a non-profit organization. Chiropractic doctors become certified in these methods to provide care to scoliosis patients. 

## Purpose

The aim of this paper was to present a detailed description of this treatment protocol as well as the theory behind it. 

## Methods

This treatment protocol consists of a combined regimen of soft tissue therapy, chiropractic manipulative therapy (CMT) and neuromuscular re-education therapy, in conjunction with a home exercise program. The goals of the protocol are to enhance motion in restricted areas of the spine, influence spinal alignment and retrain the motor sensory feedback loops involved with posture, balance and proprioception. Following are the tenets behind the application of the protocol: 

(1) Motion is essential for healthy spinal discs.

(2) Hypertonic muscles impede spinal flexibility, and hypotonic muscles reduce spinal stability.

(3) Ligamentous abnormalities contribute to proprioceptive feedback mechanisms and sensory dysfunction.

(4) Posture, proprioception, balance, and equilibrium are involuntary mechanisms, regulated by the cerebellum and automatic postural control centers.

(5) Spinal misalignments are universally present in scoliosis; CMT treats spinal misalignments and their effects upon the body.

This protocol offers a possible alternative for patients with scoliosis who have elected not to undergo bracing or surgery. However, quantitative research is needed to evaluate its effectiveness.
